# Exploring the links between alexithymia and cognitive emotion regulation strategies in internet addiction: A network analysis model

**DOI:** 10.3389/fpsyg.2022.938116

**Published:** 2022-08-01

**Authors:** Hongge Luo, Xun Gong, Xiaomei Chen, Jianing Hu, Xiaoyi Wang, Yekun Sun, Jiating Li, Shaobo Lv, Xiujun Zhang

**Affiliations:** ^1^School of Public Health, North China University of Science and Technology, Tangshan, China; ^2^College of Psychology, North China University of Science and Technology, Tangshan, China; ^3^The Department of Marxist Teaching, North China University of Science and Technology, Tangshan, China

**Keywords:** alexithymia, cognitive emotion regulation strategies, internet addiction, network analysis, college students

## Abstract

Alexithymia and emotion regulation are closely related to internet addiction. However, no research has examined how the different components of alexithymia are associated with cognitive emotion regulation in the context of multi-strategy use in internet addiction. The current study aimed to investigate the relation between alexithymia and cognitive emotion regulation in individuals with internet addiction *via* network analysis. Participants included 560 students with Young’s Internet Addiction Test scores greater than 50 points; they were also asked to complete the Toronto Alexithymia Scale (TAS-20) and the Cognitive Emotion Regulation Questionnaire (CERQ). The results revealed two bridge nodes emerging within the combined alexithymia and cognitive emotion regulation network model: “catastrophizing” and “externally oriented thoughts.” These findings indicate a more specific relation between alexithymia and cognitive emotion regulation and provide empirical evidence for targeted prevention and targeted interventions for internet addiction.

## Introduction

With the development of internet technology, the internet has become an important platform for college students to acquire knowledge, exchange ideas, and engage in leisure and entertainment. However, excessive or uncontrolled internet use may lead to “problematic internet use,” which seriously threatens the physical health, academic function, and emotional adaptation of college students (Elhai et al., 2021). Problematic internet use has become a worldwide mental health problem (Brand et al., 2020; Brand, 2022). A recent meta-analysis showed that the global pooled prevalence of internet addiction was 14.22%, and the prevalence of internet addiction increased significantly from 1999 to 2021 (Meng et al., 2022). Given the ease of internet access on college campuses, and given that college students are in the exploration stage of their identity, college students have become one of the most vulnerable groups of internet addiction (Christakis et al., 2011; Seo et al., 2019).

Previous studies have found that internet addiction was associated with multiple factors, including socio-demographic (Sela et al., 2021), neurobiological (Paik et al., 2017), cognitive (Antons et al., 2020), personality (Mak et al., 2021), social (Hao et al., 2022), and family (Chung et al., 2019) characteristics. Negative emotions are critical factors in maintaining and exacerbating addiction and in relapse after withdrawal (Baker et al., 2004), so factors associated with negative emotions are very important in understanding addiction. People with alexithymia, a condition associated with difficulty identifying one’s emotions, tend to have a greater tendency to produce more negative emotions. Therefore, this study examined the relationship between alexithymia, emotion regulation, and internet addiction.

### Internet addiction and alexithymia

Alexithymia, which was first found in clinical patients with psychosomatic disease, is a kind of basic and stable personality characteristic involving emotional–cognitive aspects. It is characterized by an individual’s difficulty in identifying emotions and expressing them using appropriate language, lack of fantasy, extroverted thinking, and lack of ability to reveal one’s internal attitudes, feelings, and desires (Nemiah and Sifneos, 1970; Bagby and Taylor, 2009; Taylor and Bagby, 2021). Alexithymia predisposes individuals to a variety of physiological and psychiatric disorders (Bagby and Taylor, 2009; Luo et al., 2021). Alexithymia may also be important in pathogenesis of substance use disorders, behavioral addiction (Bonnaire et al., 2013; Morie et al., 2016), and internet addiction (El-Rasheed et al., 2017; Luo et al., 2022). Previous studies have found a significant positive relation between internet addiction and alexithymia among high school students (Scimeca et al., 2014; di Nicola et al., 2017; Bolat et al., 2018; Karaer and Akdemir, 2019; Lin, 2020), college students (Dalbudak et al., 2013; Kandri et al., 2014), and adults (Bipeta et al., 2015; Scimeca et al., 2017).

Individuals with alexithymia cannot properly describe their emotions and understand the reasons for others’ emotional changes, rendering a negative impact on interpersonal relationships, such as social difficulties and lack of confidence (Gross, 2010; Besharat and Shahidi, 2011). Given the ubiquity and convenience of the internet, individuals can rely on it to exchange information with others, producing a virtual social mode to replace social communication in real life (Morahan-Martin and Schumacher, 2000). The regular use of such internet tools or virtual social media may also lead to the occurrence of real-life social isolation and more serious problems of internet use behaviors (Young, 1998).

### Internet addiction and cognitive emotion regulation

There are two main types of strategies for cognitive emotion regulation, which is the regulation of emotional experience and expression by consciously changing thoughts or cognition when responding to negative or stressful events: adaptive strategies and maladaptive strategies (Garnefski et al., 2004). Adaptive strategies, including acceptance, positive refocusing, refocusing on planning, positive reappraisal, and putting into perspective, can enable individuals to adapt to and deal with the current situation and avoid the occurrence of various adverse psychological outcomes. Maladaptive strategies, including self-blame, rumination, catastrophizing, and blaming others, may affect individuals’ response and adaptability to social stimuli, thus triggering negative emotions (Garnefski et al., 2001).

Individuals with emotional dysregulation are prone to risky and compulsive behaviors and maladaptive coping strategies for managing negative emotions (Weiss et al., 2015). Emotion regulation plays a crucial role in internet addiction (Mo et al., 2018; Quaglieri et al., 2022). Individuals with problematic internet use show difficulties in accepting negative emotions, controlling impulsive behaviors, and accessing effective emotion regulation strategies (Pettorruso et al., 2020). Some studies confirmed that individuals who are more likely to use maladaptive strategies may overuse the internet to suppress or compensate for negative feelings and thoughts (Extremera et al., 2019; Balıkçı et al., 2020; Gioia et al., 2021). Furthermore, a 12-year longitudinal study showed a strong direct link between unbalanced emotion regulation in infancy and internet addiction in adolescence (Cimino and Cerniglia, 2018).

### Alexithymia and cognitive emotion regulation

Alexithymia reflects deficits in the regulatory processes of emotion and cognitive processing (Luminet et al., 2021; Taylor and Bagby, 2021). Cognitive emotion regulation reflects both the cognitive and regulatory characteristics of emotions. Some people with alexithymia adopt less positive and more negative cognitive regulation strategies (Chen et al., 2011; Dorosheva, 2020). Given the complexity and flexibility of emotion regulation, individuals tend to adopt multiple strategies (whether positive or negative) to regulate emotions when facing negative events, and various strategies may have complex interactions (Brans et al., 2013). Thus, assessing only the use of a single strategy in isolation cannot reveal the relation between alexithymia and cognitive emotion regulation.

Furthermore, different structural components of alexithymia have different effects on emotion regulation, and a high level of recognition is associated with ascribing emotional difficulties with worse emotional expression. Notably, some individuals with alexithymia with higher extroverted thinking scores are relatively effective in emotion regulation (Chen et al., 2011). Therefore, it is necessary to explore the relationship between the different dimensions of alexithymia and regulation strategies in the context of multi-strategy use.

### Internet addiction, alexithymia, and cognitive emotion regulation

The Interaction of Person-Affect-Cognition-Execution (I-PACE) model is a comprehensive theoretical model for explaining the development and maintenance of addictive behaviors (Brand et al., 2014, 2016, 2019). The model includes the following main components: (1) the P component of the model refers to the core characteristics of person (including biopsychological constitution, personality, social cognitions, psychopathology, and motives); (2) the A and C components of the model refer to affective and cognitive responses to internal or external triggers (including coping, internet-related cognitive biases, mood regulation, and attentional biases); (3) the E component of the model refers to execution functions dominated by the prefrontal cortex (including general inhibitory control, inhibitory control of stimuli-specific, and decision making). The consequences of using the internet refers to the experience of satisfaction and compensation associated with internet use behavior and the transition from voluntary and impulsive behavior to more habitual and compulsive behavior over time.

The model emphasizes the development of addictive behavior as a result of interactions between these components. Specifically, this model proposes that the P-component dimensions are predisposing variables for excessive internet use, which are fundamental, and the A and C components are determinants, which are regulators and mediators that can influence the decision of whether to use the internet. Due to cue-reactivity, craving, low inhibitory control, and seeking immediate rewards, the E component causes individuals to make adverse decisions to use the internet again (Antons et al., 2020), and individuals gain satisfaction and compensation after internet use, which in turn affects the dynamic processes of the above components, such as positive reinforcement of poor coping styles and internet use expectancies and negative reinforcement of a person’s core characteristics. Eventually, this progression forms a circular and dynamic process.

Brand et al. (2019) further refined the I-PACE model to divide internet overuse into early and late stages. Brand (2022) then proposed two driving paths for internet addictive behaviors, namely the “feels better” path and the “must do” path. The “feels better” path refers to how the internet alleviates the individual’s negative emotions and makes the individual feel better. The “must do” path refers to how the behavior of using the internet gradually becomes habitual and compulsive behavior. In the early stage, the “feels better” path emphasizes the importance of interactions between personal background factors and affective and cognitive responses in the occurrence of addictive behavior. In the later stage, the “must do” path is involved based on the “feels better” path, and stimuli-specific reductions in inhibitory control and compensation play an important role in the maintenance of addictive behaviors. We focused more on the “feels better” path of internet overuse. Alexithymia, a relatively stable personality trait, belongs to a dimension of the P component. Mood regulation plays a significant role in the development of internet addiction, and cognitive emotion regulation falls under a dimension of the A and C components. The interaction of the P component and the A and C components has different effects on individual behaviors. Therefore, addressing the mechanism of their interaction has important implications for prediction of and intervention for internet addiction.

### Study aims and hypotheses

Network analysis is the use of psychological networks to describe the complex interactions among variables. Network analysis has two main components: “nodes,” representing the observed variables, and “edges,” representing the statistical relation between the observed variables (Borsboom, 2008). The indices of each node in the network are calculated to compare their importance. Our study aimed to explore how alexithymia interacts with cognitive emotion regulation strategies in individuals with internet addiction using network analysis, that is, we aimed to determine which key nodes connect the two factors. We expected our findings to provide a theoretical basis for targeted interventions for internet addiction. Given that network analysis is an exploratory technique describing complex interactions between variables, we formulated no hypotheses on the specific variables that could appear as central and bridge nodes in these network models.

## Materials and methods

### Participants

We used cluster sampling to recruit 1,677 students from North China University of Science and Technology between November and December 2021. Among them, 560 participants with Young’s Internet Addiction Test (IAT) score higher than 50 points, indicating that they were prone to internet addiction, were selected. Their sociodemographic variables were assessed, including time spent on the internet, sex, age, grade level, aspects of alexithymia, cognitive emotion regulation strategies, and internet addiction *via* an electronic structured questionnaire. To ensure the reliability and confidentiality of the data, we administered the questionnaires anonymously. The participants (males, 317 [56.4%]) were aged between 18 and 26 years (20.52 ± 1.41). Of the participants, 160 (28.57%), 242 (43.21%), 66 (11.79%), and 92 (16.43%) were in the first, second, third, and fourth year of college, respectively. The participants reported spending an average of 7.73 ± 4.74 h online per day. The entire dataset of 1,677 can be found in the Supplementary Table 1.

### Ethics

The study protocol was conducted based on the Declaration of Helsinki and was approved by the research ethics board of the North China University of Science and Technology on February 27, 2021 (No. 2021020).

### Measures

#### Internet addiction

We used the Chinese version of Young’s IAT (Young, 1998; Jianqin et al., 2010) to screen internet users for addiction, addiction level, and impact of the internet on life. The tool has 20 items that are rated on a five-point scale from 1 (*almost never*) to 5 (*always*; e.g., “How often do you feel depressed, moody, or nervous when you are off-line, which goes away once you are back online?”). The scores for each item are added to get the total score. A total score of >50 points indicates a possible internet addiction problem. Cronbach’s α for the Chinese version of the IAT was 0.91, and the criterion validity was 0.71.

#### Alexithymia

We measured alexithymia using the Chinese version of the Toronto Alexithymia Scale (TAS-20) (Yi et al., 2003). The scale has 20 items and contains three factors: Difficulty Identifying Feelings (DIF, e.g., “I am often confused about what emotion I am feeling”), Difficulty Describing Feelings (DDF, e.g., “It is difficult for me to reveal my innermost feelings, even to close friends”), and Externally Oriented Thoughts (EOTS, e.g., “I prefer to analyze problems rather than just describe them”). Using a five-point scale, participants reported responses from 1 (*totally disagree*) to 5 (*totally agree*). A higher score indicates greater alexithymia (Bagby et al., 1994). The test–retest reliability and Cronbach’s α for the Chinese version were 0.87 and 0.83. It has been shown to be a good mood barrier assessment tool in China.

#### Cognitive emotion regulation

We used the Cognitive Emotion Regulation Questionnaire-Chinese Version (CERQ-C) (Zhu et al., 2007) to evaluate the use of cognitive emotion regulation strategies of the participants when experiencing negative events. Responses are reported using a five-point scale from 1 (*almost never*) to 5 (*almost always*). The CERQ-C contains nine dimensions: acceptance, positive refocusing, refocusing on planning, positive reappraisal, putting into perspective, self-blame, rumination, catastrophizing, and blaming others. Of these, the first five belong to adaptive strategies and the latter four belong to maladaptive strategies. Each dimension consists of four items, for a total of 36 items (e.g., “I feel that I am the one to blame for it”). The entry score of each dimension gives the subscale score. The higher the score in a certain dimension, the more likely the subject tends to adopt this specific cognitive emotion regulation strategy when coping with negative events (Garnefski et al., 2001). The Cronbach’s α and the test–retest reliability of the scale was 0.81 and 0.56, showing appropriate reliability and validity. It is a reliable tool for assessing cognitive strategies for emotion regulation.

### Analysis plan

#### Network analysis

An advantage of network analysis is the ability to adopt Graphical Least Absolute Shrinkage and Selection Operator (GLASSO). GLASSO is the introduction of penalization factors based on the partial correlated network model to remove connections with a relatively weak degree of connection in the network. By reducing the number of connections, the model can fit a network structure that is more easily explained by researchers and more predictively accurate (Pourahmadi, 2011). The three centrality indicators of the GLASSO network (i.e., strength, between, and closeness) are calculated to compare the importance of each node in the network. Strength centrality is the sum of the weighted values of all connections at a node, used to measure the importance of nodes in a network. Between centrality is the frequency of a node on the shortest path of any other two nodes. Closeness centrality is the reciprocal of the average shortest path length between a node and other nodes. Closeness and between measure the importance of nodes to the overall information transmission of the network (Costantini et al., 2015).

Another advantage of network analysis is its ability to identify bridging variables, which represent a direct association for the development and maintenance of two psychological problems, thus making the intervention more targeted. In addition, networks are characterized by predictability, which indicates the extent to which variation at a node can be predicted by a variation connected to it. The average predictability of all the nodes in a network reflects the degree to which the network is affected by the factors outside the network. A high average predictability means that the network structure can predict each factor well internally, and the external factors can explain the variation (Haslbeck and Waldorp, 2018).

We performed network analysis with R 4.0.0 software. First, we used a partial correlation network model between the three factors of alexithymia and the nine cognitive emotion regulation strategies using the *qgraph* package (Epskamp et al., 2012). For a partial correlation network with 12 nodes, 66 parameters (12 × 11/2) need to be estimated, with a minimum of three to five individuals per parameter (Epskamp et al., 2018). Our sample size was 560 people, which was sufficient to guarantee the reliability of the data analysis. Based on the partial correlation network, we adopted GLASSO to remove weak connections and obtain a concise network. The predictability of nodes was estimated using the *mgm* package.

#### Network centrality

Network centrality represents how much, how strongly, and how closely a node is connected to other nodes. Here, degree centrality, between centrality, and closeness centrality reflected the importance of a node in the network. The correlation stability (CS) coefficient was calculated using the *bootnet* package of R software with 1,000 case-dropping bootstraps to assess the stability of centrality indices; higher CS coefficients indicated a stronger stability of the centrality indices (Epskamp and Fried, 2018). A previous study has suggested that a CS coefficient ≥0.50 indicates sufficient stability of the centrality indices (Epskamp et al., 2018).

#### Bridge centrality

We computed the bridge centrality index, or the Bridge Expected Influence (BEI), of the GLASSO network using the *networktools* package of R software to assess which nodes could “bridge” alexithymia and cognitive emotion regulation communities (Jones et al., 2021). Bridge expected influence indicates the sum of the connections of one node with the other nodes of another community. Higher scores indicated that a node was more closely connected to all the nodes in the other groups. More specifically, the bridge nodes were determined by comparing the BEI value in different dimensions within the community, and the node with the largest BEI is the bridge node of the community. R codes for network analysis can be found in the Supplementary Table 2.

## Results

### Network estimation

**FIGURE 1:**
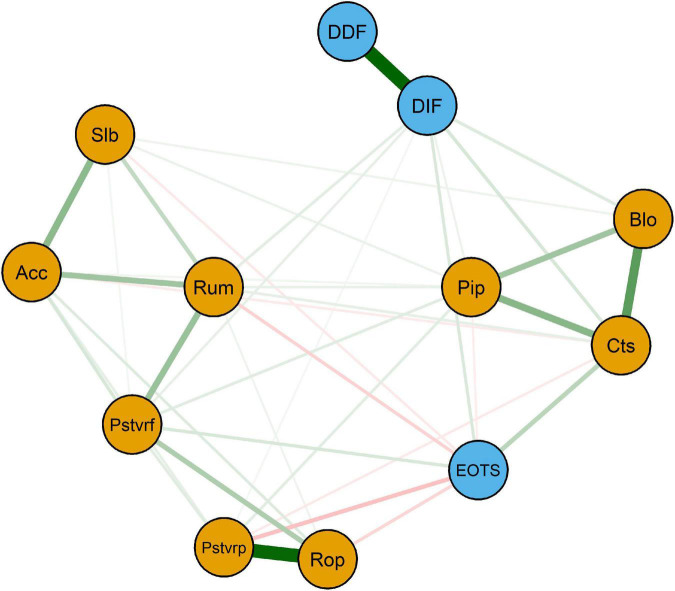
Regularized partial correlation network: Network structure of the TAT and CERQ-C, based on the whole sample (*n* = 560). Each subscale is represented by a node. Green connections represent positive associations, whereas red connections represent negative associations. Thicker edge (positive and negative) signifies stronger partial correlations. Slb, Self-blame; Acc, Acceptance; Rum, Rumination; Pstvrf, Positive refocusing; Rop, Refocusing on planning; Pstvrp, Positive reappraisal; Pip, putting into perspective; Cts, Catastrophizing; Blo, Blaming others; DIF, Difficulty Identifying Feelings; DDF, Difficulty Describing Feelings; EOTS, Externally-Oriented Thoughts.

Figure 1 illustrates the estimated network. Among the three factors of alexithymia, DIF and DDF (0.68) showed the strongest connection weight. Among the nine strategies, the strongest weights were between refocusing on planning and positive reappraisal (0.62), followed by catastrophizing and blaming others (0.46), putting into perspective and blaming others (0.30), and catastrophizing and putting into perspective (0.23); all were positively related. The between-groups connection was weaker than the within-group one. Catastrophizing was positively associated with EOTS (0.20) and DIF (0.11). EOTS was negatively associated with positive reappraisal (−0.18) and rumination (−0.14). The weights of all connections in the GLASSO network are shown in Table 1. In addition, the average predictability of all nodes in the network was *R*^2^ = 0.63.

**TABLE 1:** Weights of the connections in the Graphical Least Absolute Shrinkage and Selection Operator (GLASSO) network.

	1	2	3	4	5	6	7	8	9	10	11	12
1. Slb	0											
2. Acc	0.31	0										
3. Rum	0.05	0.21	0									
4. Pstvrf	0	0.19	0.24	0								
5. Rop	0.14	0.11	0.19	0.14	0							
6. Pstvrp	0	0	0	0.19	0.62	0						
7. Pip	0.05	0	0.05	0.06	0	0.08	0					
8. Cts	0	0	0.12	0	0	0	0.23	0				
9. Blo	0	0	0	0	0	0	0.30	0.46	0			
10. DIF	0	0	0	0.07	0	0	0	0.11	0.07	0		
11. DDF	0	0	0	0	0	0	0	0	0	0.68	0	
12. EOTS	0	0	0.14	0.09	0	0.18	0	0.2	0	0.10	0	0

Slb, Self-blame; Acc, Acceptance; Rum, Rumination; Pstvrf, Positive refocusing; Rop, Refocusing on planning; Pstvrp, Positive reappraisal; Pip, putting into perspective; Cts, Catastrophizing; Blo, Blaming others; DIF, Difficulty Identifying Feelings; DDF, Difficulty Describing Feelings; EOTS, Externally-Oriented Thoughts.

### Centrality estimation

**FIGURE 2:**
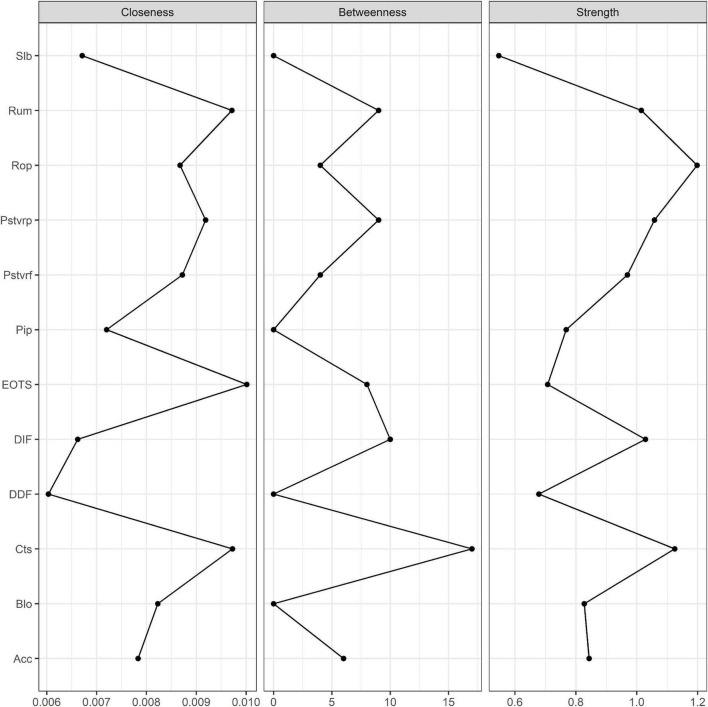
Centrality plot for the regularized network. Strength measures the importance of nodes in a network. Closeness and Between measure the importance of nodes to the overall information transmission of the network. Slb, Self-blame; Acc, Acceptance; Rum, Rumination; Pstvrf, Positive refocusing; Rop, Refocusing on planning; Pstvrp, Positive reappraisal; Pip, putting into perspective; Cts, Catastrophizing; Blo, Blaming others; DIF, Difficulty Identifying Feelings; DDF, Difficulty Describing Feelings; EOTS, Externally-Oriented Thoughts.

Figure 2 shows the centrality estimates. EOTS had the highest closeness, followed by catastrophizing and rumination, suggesting that their influence could spread more quickly to other nodes. Catastrophizing had the highest betweenness, followed by DIF, rumination, and EOTS, suggesting that these could play an intermediary role in the information transmission of the network. Refocusing on planning had the highest strength in the network, followed by catastrophizing, positive reappraisal, and DIF, suggesting their higher importance in the network. The *CS* coefficient (*CS* [cor = 0.7] = 0.75) indicated that the node centrality index of the network had good stability.

### Bridge centrality

**FIGURE 3:**
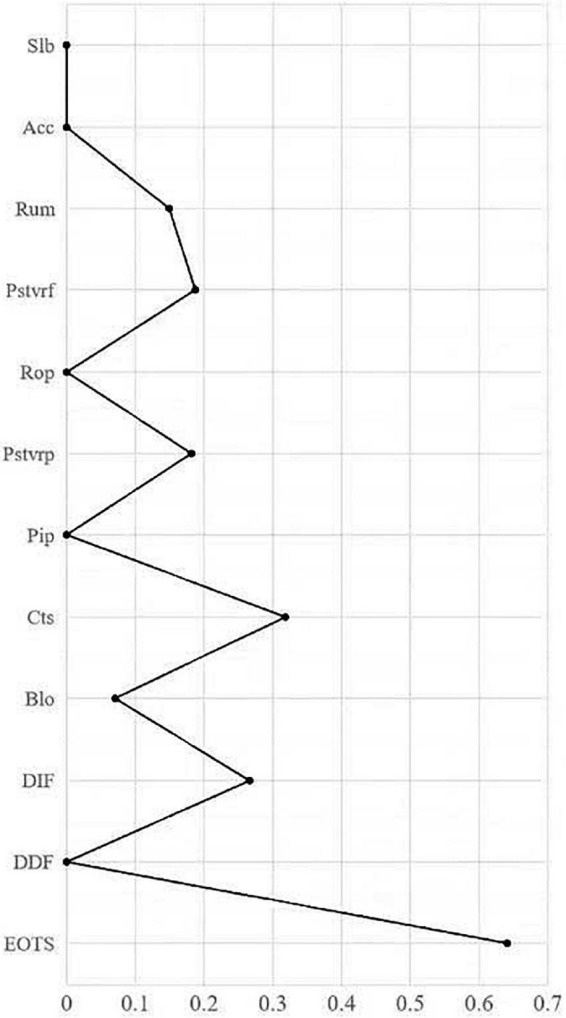
Bridge Expected Influence (1-step). Slb, Self-blame; Acc, Acceptance; Rum, Rumination; Pstvrf, Positive refocusing; Rop, Refocusing on planning; Pstvrp, Positive reappraisal; Pip, putting into perspective; Cts, Catastrophizing; Blo, Blaming others; DIF, Difficulty Identifying; DDF, Difficulty Describing Feelings; EOTS, Externally-Oriented Thoughts.

Figure 3 shows the bridge centrality indicators. In the network model, EOTS has the largest BEI value in the alexithymia community, and catastrophizing has the largest BEI value in the cognitive emotion regulation strategies community, thus EOTS and catastrophizing emerged as bridge nodes.

## Discussion

We used network analysis to explore the relation between different components of alexithymia and different cognitive emotion regulation strategies in the context of multi-strategy use in individuals with internet addiction. Catastrophizing and EOTS were shown to play a large role in the network model. The network estimation results showed that the connection between catastrophizing and EOTS had the largest between-group weight, indicating that the two were the most closely related among all the nodes. The closeness and between centrality values of these two nodes were very high, indicating that they also had a significant effect in the overall information transmission of the network. Moreover, they not only affected other nodes but also transmitted the influence of other nodes. The bridge centrality results also showed that catastrophizing and EOTS were two bridge nodes, suggesting that EOTS from the alexithymia cluster may have a great influence on the choice of strategy, and catastrophizing from the cognitive emotion regulation strategies cluster may play a strengthening role in alexithymia.

People with alexithymia perceive more negative emotions (Franzoi et al., 2020) and stress (de Timary et al., 2008). The EOTS factor is negativity and inability in the face of negative emotions and stressors (Moriguchi et al., 2007). Catastrophizing is a negative cognitive emotion regulation strategy that exaggerates the severity and overestimates the potential threat of negative emotions and stress (de Timary et al., 2008; Hao-Xian et al., 2018). This distortion compels individuals to pay more attention to their emotional states rather than to reducing the intensity of pain, and makes them more likely to have more negative emotions (Kaczkurkin and Foa, 2015). Subsequently, the greater the negative emotions, the more likely they are to make risky decisions. Indeed, people with alexithymia tend to choose less valuable immediate rewards and abandon more valuable delayed rewards (Guan et al., 2015). Thus, they can easily form false expectations that spending time on the internet may effectively alleviate their negative emotions, at least temporarily. Once the negative mood and stress are relieved, gratification occurs, which strengthens the use of maladaptive cognitive emotion regulation strategies and cognitive bias toward the internet. At this point, the use of the behavior is voluntary and impulsive.

Emotion regulation plays a critical role in the formation of addiction. According to the principle of conditioned reflex, individuals connect the relieved positive emotions with network clues, resulting in a craving for the internet. To alleviate the craving, individuals will further adopt the regulation method of catastrophizing and use the internet more. The negative consequences of internet overuse, such as loneliness, conflicts with family and friends, misunderstood feelings, emptiness, and other negative emotions and experiences, will increase (King and Delfabbro, 2018). Gratification becomes less important, as compensation becomes more important. At this point, the internet use behavior changes from voluntary and impulsive to habitual and/or compulsive. In addition, individuals who score high in EOTS lack internal awareness (Rinaldi et al., 2017) and internal monitoring (Correro et al., 2021). They tend to have rigid thought patterns—once strategies are chosen, adjusting becomes difficult.

We also found that the between and closeness centralities of rumination were stronger than other nodes, indicating that rumination plays an important role in the overall information transmission of the network. Rumination is a maladaptive adjustment strategy in which an individual continually thinks about feelings and thoughts associated with negative events (Garnefski et al., 2001). Moreover, the strength of refocusing on planning and positive reappraisal was stronger, and the two were positively correlated, whereas positive reappraisal was negatively correlated with EOTS. Individuals with high EOTS are less likely to choose these two positive regulation strategies, which in turn leads to worse emotion regulation and state (Dorosheva, 2020). All of these strategies can worsen negative emotions, consequently increasing the possibility of overusing the internet as a maladaptive pattern (Spada et al., 2015).

The correlation among catastrophizing, blaming others, and putting into perspective was high. Thus, individuals who often use catastrophizing were more likely to choose to blame others and put into perspective at the same time. Catastrophizing is a negative strategy, whereas rational analysis is a positive strategy. When faced with emotion-eliciting stimuli, individuals may be unsatisfied with implementing only one strategy to regulate emotional results; they may attempt other strategies, such that positive and negative strategies may be selected (Aldao and Nolen-Hoeksema, 2013). Therefore, when individuals with internet addiction regulate their emotions in a catastrophized manner in the face of negative events, they will attempt to blame events on others or compare events with other events to find positive components to relieve their negative emotions. However, the use of multiple strategies to cope with stimuli may imply a misleading attempt at emotion regulation. Positive and negative strategies are selected at the same time, but individuals with more numerous types and higher levels of negative strategies have worse mental health (Burke et al., 2018). These individuals may then seek to relieve negative emotions by overusing the internet as a poor way to cope. We also investigate the relation between alexithymia and cognitive emotion regulation in individuals without internet addiction *via* network analysis. The results of the network analysis show that the links between alexithymia and cognitive emotion regulation are different in those with and without internet addiction (More details in Supplementary Table 3).

### Implications of the study

This study offered several important implications for future clinical intervention. Our findings highlighted EOTS and catastrophizing as two critical bridge nodes between cognitive emotion regulation strategy and alexithymia in individuals with internet addiction. First, our study may contribute to the formulation of targeted prevention. Unsafe attachment, lack of family communication, and indifferent parenting style may be risk factors for alexithymia (Thorberg et al., 2016). For high-risk groups prone to internet addiction, such as those with high scores in the EOTS subscale, corresponding psychological intervention measures could be taken from the aspects of attachment relationships, interpersonal relation, coping style, and emotion perception.

Second, the study has implications for carrying out targeted treatment. The I-PACE model indicates that poor cognition (false expectations of the internet) and emotion (satisfaction/compensation) are the key factors in addictive behavior, while poor cognitive strategies (e.g., overstating and overestimating the severity of events) reinforce them. Individuals gradually establish conditioned reflexes between the internet and positive emotions. Behavioral therapy can be provided to eliminate this conditioned reflex. Catastrophizing strategies make individuals pay more attention to their emotional state than to pain reduction. Mindfulness is a state of consciousness produced by purposeful and non-judgmental attention to the present; it focuses on learning in the face of depression and negative thinking, and emphasizes the conscious need to point to current experiences and maintain an open, curious, and receptive attitude (Bishop et al., 2004). Mindfulness can effectively improve the symptoms of internet addiction (Rosenthal et al., 2021), and this improvement is long-term (Calvete et al., 2017). Therefore, mindfulness therapy can be used to improve poor cognitive strategies and to treat internet addiction effectively.

### Limitations and future research

There were some limitations in the study. First, a cross-sectional design was used, and as such, the causal relationship between cognitive emotion regulation and alexithymia could not be obtained. Longitudinal tracking and experimental methods can be carried out to clarify the potential causal linkage of alexithymia and cognitive emotion regulation strategies in the case of internet addiction.

Second, there are different types of internet addiction, which were not distinguished in this study. The I-PACE model points out that different predisposing variables and specific hobbies affect different types of internet addiction (Brand et al., 2019). Future studies could attempt to identify whether the complicated relation between different cognitive emotion regulation strategies and alexithymia can have a different effect on different types of internet addiction.

## Conclusion

This study revealed two crucial nodes, catastrophizing and EOTS, in the combined alexithymia and cognitive emotion regulation network model. The two nodes have a high importance in the information transfer of the network model, and their influence spreads to other nodes quickly. The interaction of EOTS, catastrophizing, and other strategies causes individuals to produce and exaggerate negative emotions. Some of these individuals use the internet problematically to bring temporary and superficial pleasure to relieve negative emotions, and after repeated reinforcement and conditioning, they gradually form addictive behavior.

## Data availability statement

The original contributions presented in this study are included in the article/Supplementary material, further inquiries can be directed to the corresponding author.

## Ethics statement

The studies involving human participants were reviewed and approved by the Research Ethics Boards of the North China University of Science and Technology. The patients/participants provided their written informed consent to participate in this study.

## Author contributions

HL and XZ designed the experiment. XC, JH, XW, YS, and JL collected the data. XG and SL analyzed the data. HL wrote the original draft. All authors contributed to the article and approved the submitted version.
