# Supplementary material for: Exploring the links between alexithymia and cognitive emotion regulation strategies in internet addiction: A network analysis model
Source: Front Psychol. 2022 Aug 1;13:938116. doi: 10.3389/fpsyg.2022.938116 (PMC9376254; doi:10.3389/fpsyg.2022.938116)
Supplement: Supplementary file 2 [file Table_2.DOCX]

Supplementary 2: The code for R software.

**#安装包调用（intall package）**

library("foreign")

library("IsingSampler")

library("Hmisc")

library("IsingFit")

library("qgraph")

library("bootnet")

library("networktools")

library("haven")

library("Rcpp")

library("corpcor")

library("ggplot2")

library("mgm")

**#载入Excel数据 （load data）**

data1<-read.csv("XXXXXX.csv")

attach(data1)

**#数据命名** **（data naming）**

short.names<-c("Slb","Acc","Rum","Pstvrf","Rop","Pstvrp","Pip","Cts","Blo","DIF","DDF" ,"EOTS")

long.names<-c("自我责难","接受","沉思","积极重新关注","重新关注计划","积极重新评价","理性分析","灾难化","责难他人","情感辨别不能","情感描述不能","外向型思维")

groups<-list("认知情绪调节策略"=c(1,2,3,4,5,6,7,8,9),"述情障碍"=c(10,11,12))

colour<-list("认知情绪调节策略"=c(1,2,3,4,5,6,7,8,9),"述情障碍"=c(10,11,12))

**#模型运算（model operation）**

fit_mgm<-mgm(data1,type=c("g","g","g","g","g","g","g","g","g","g","g","g"),levels=c(1,1,1,1,1,1,1,1,1,1,1,1),layout="spring",labels=short.names,nodeNames=long.names,groups=colour,cut=0,vsize=6,border.width=1.5,theme="colorblind",legend.cex=.40,k=2,lambdaSel="CV",lambdaFolds=10,ruleReg="AND")

**#边缘连接强度计算 （Edge strength）**

round(fit_mgm$pairwise$wadj,3)

**#网络图绘制（Network diagram drawing）**

Network1<-qgraph(fit_mgm$pairwise$wadj,layout="spring",repulsion=1,edge.color=fit_mgm$pairwise$edgecolor,labels=short.names,nodeNames=long.names,groups=colour,legend.mode="style2",legend.cex=0.5,vsize=6,esize=10)

**#网络图导出tiff格式（export Network.tiff）**

tiff("2.tiff",,width=6.5,height=4,units="in",res=800,compression="lzw")

qgraph(Network1,layout="spring",labels=short.names,nodeNames=long.names,groups=colour,cut=0,vsize=6,border.width=1.5,theme="colorblind",negDashed=TRUE,legend.cex=.4)

dev.off()

**#节点中心性指标运算(Centrality)**

centrality<-centrality_auto(Network1,weighted=TRUE,signed=TRUE)

nc<-centrality$node.centrality

SPL<-centrality$ShortestPathLengths

nc

SPL

**#中心性指标折线图导出tiff格式(export Centrality.tiff)**

tiff(file="1.tiff",width=8,height=8,units="in",res=800,compression="lzw")

qgraph::centralityPlot(Network1,include=c("Closeness","Betweenness","Strength"),scale="raw")

dev.off()

**#桥梁中心性指标运算(Bridge Centrality)**

A<-qgraph(Network1,labels=short.names,layout="spring",vsize=6,cut=0,border.width=1.5,border.color="black",groups=groups,color=c("#FFFACD","#D7BDE2","#AED6F1","#DAF7A6","#F5B7B1"),nodeNames=long.names,legend.cex=.4)

B<-bridge(A,communities=groups,directed=FALSE)

B

**#节点可预测性计算(Predictability)**

pred_mgm<-predict(object=fit_mgm,data=data1,errorCon=c("RMSE","R2"),errorCat=c("CC","nCC"))

pred_mgm$errors
