# Supplementary material for: Exploring the links between alexithymia and cognitive emotion regulation strategies in internet addiction: A network analysis model
Source: Front Psychol. 2022 Aug 1;13:938116. doi: 10.3389/fpsyg.2022.938116 (PMC9376254; doi:10.3389/fpsyg.2022.938116)
Supplement: Supplementary file 3 [file Table_3.DOCX]

Supplementary3: the results and interpretations of the network analysis for the non-internet addiction group.

We investigate the relation between alexithymia and cognitive emotion regulation in individuals without internet addiction via network analysis to further inform targeted interventions for internet addiction. The results of the network analysis show that the links between alexithymia and cognitive emotion regulation are different in those with and without internet addiction, as described next.

This issue about identifying the non-addiction group is first explained. A total score of Young’s Internet Addiction Test (IAT) ＞50 points indicated a possible internet addiction problem (Cao Jianqin et al., 2010). Thus, 560 students with IAT score higher than 50 points were selected as internet addiction group in this study. If all subjects with scores below 50 points are analyzed as the non-addiction group, the results may be confounding. First, The IAT assesses the risk of internet addiction, rather than a diagnosis of internet addiction. The IAT total score ranges, with the higher the score representing the higher risk of internet addiction. And the IAT manual states that a total scores from 31 to 49 is close to 50 points, indicating the presence of a mild level of internet addiction. Second, there are a total of 712 individuals in the range of 31~49 points (31~40 points: 392; 41~49 points: 320). The sample is relatively large, which will have a great impact on the results, especially the samples between 41~49 points. Last, the IAT manual also states that a total score <30 points are considered to reflect a normal level of internet usage. Therefore, the 251 samples with scores below 30 points, representing the non-internet addiction group, were analyzed for clearer results

Figure1-3 show the network analysis results of the non-internet addiction group. Figure 4-6 show the network analysis results of the internet addiction group.


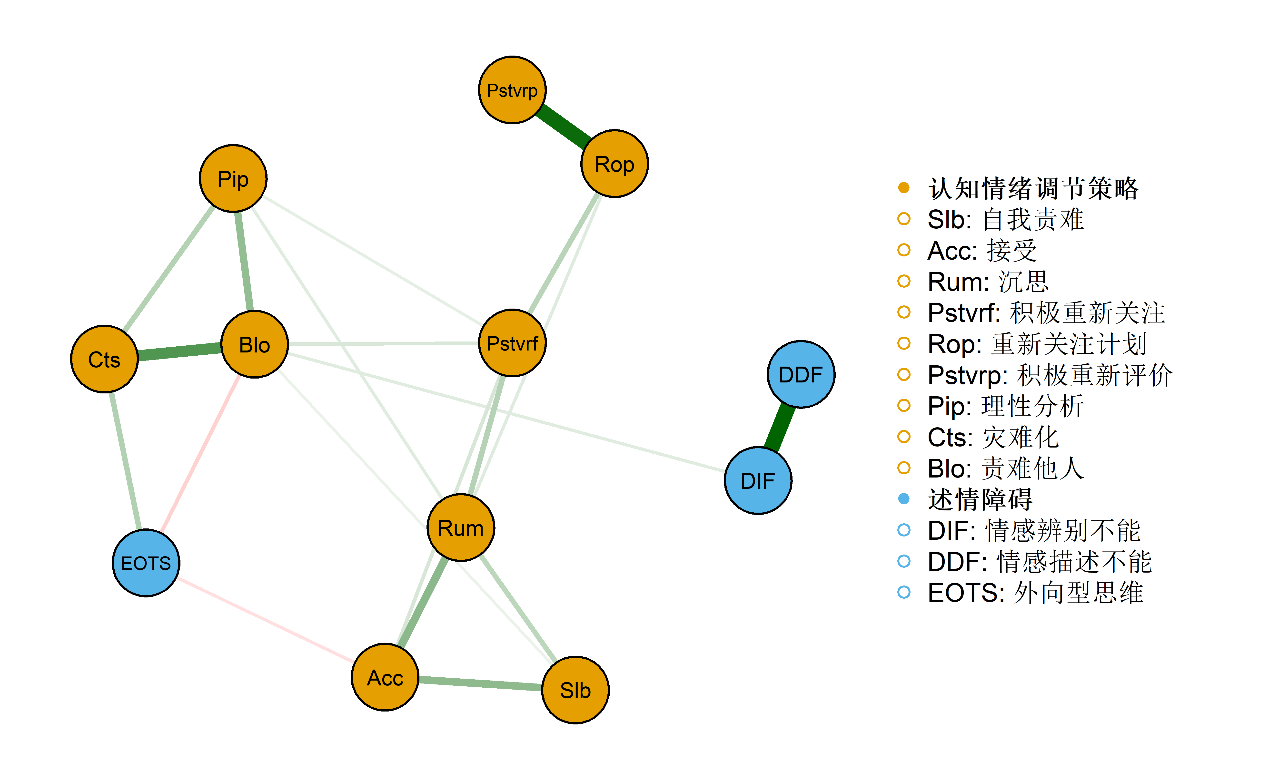


Figure 1 Regularized partial correlation network of the non-internet addiction group (n = 251).

Nodes: Slb, Self-blame; Acc, Acceptance; Rum, Rumination; Pstvrf, Positive refocusing; Rop, Refocusing on planning; Pstvrp, Positive reappraisal; Pip, putting into perspective; Cts, Catastrophizing; Blo, Blaming others; DIF, Difficulty Identifying Feelings; DDF, Difficulty Describing Feelings; EOTS, Externally-Oriented Thoughts. The following abbreviations are the same as these.


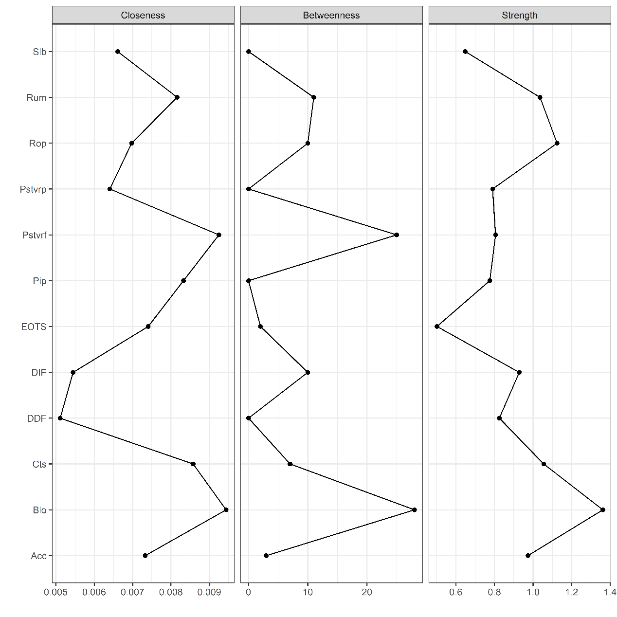

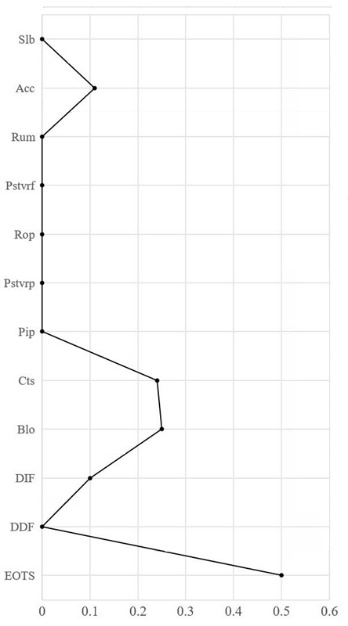


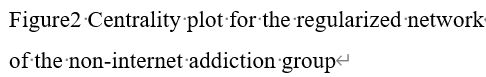

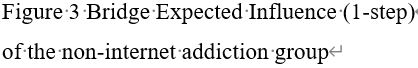


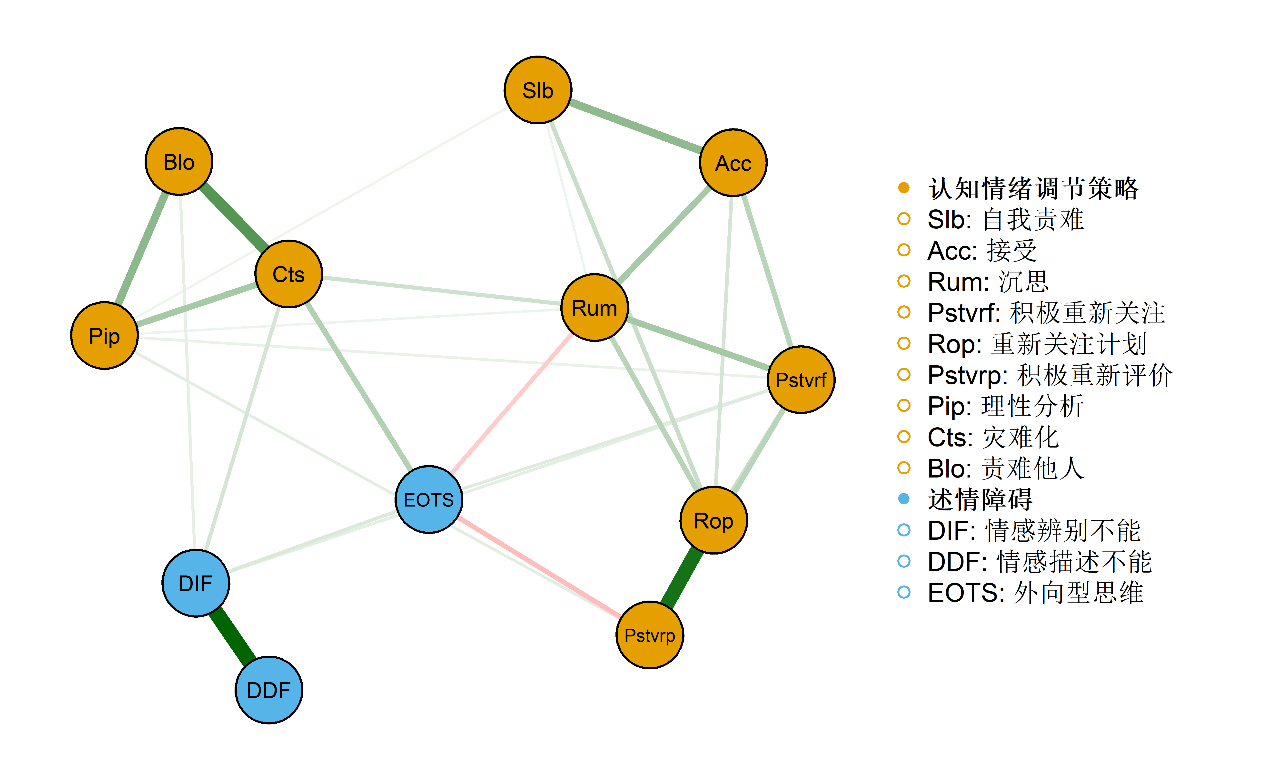


Figure 4 Regularized partial correlation network of the internet addiction group.


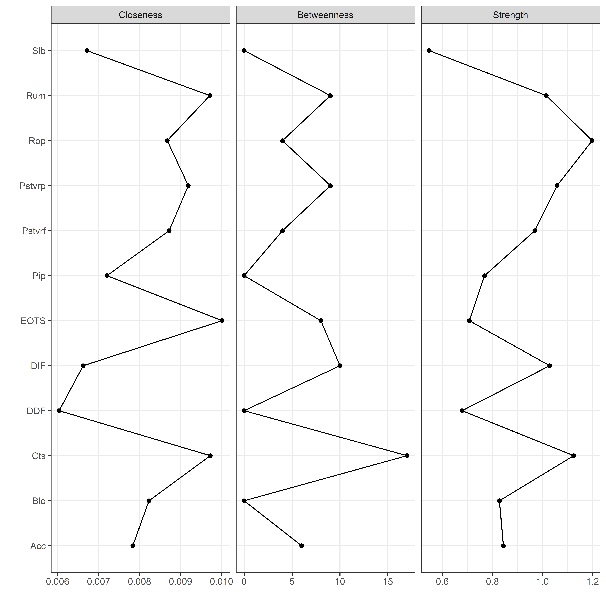

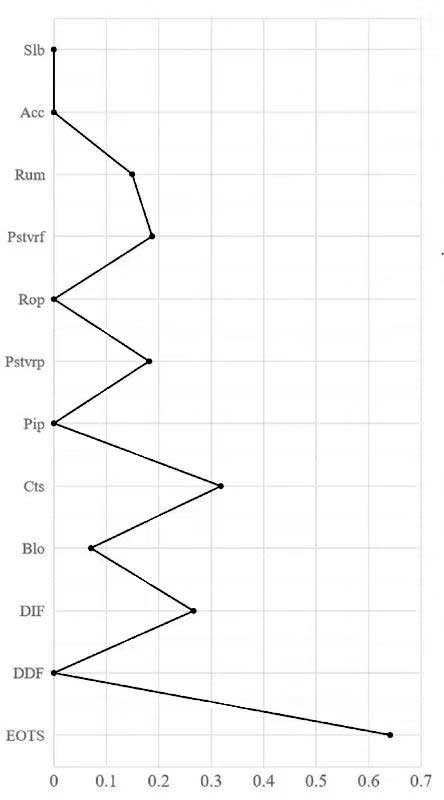


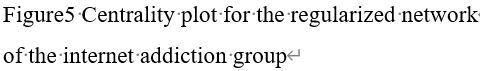

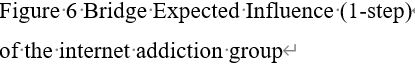


Table1 Results of the network analysis for the two groups

| non-internet addiction group  (the IAT scores＜30, n=251) | internet addiction group  (the IAT scores≥50, n=560) |
| --- | --- |
| Closeness Centrality | Closeness Centrality |
| Positive refocusing | Externally-Oriented Thoughts |
| Blaming others | Rumination |
| Between Centrality | Between Centrality |
| Positive refocusing | Catastrophizing |
| Blaming others | Difficulty Identifying Feelings |
| Strength Centrality | Strength Centrality |
| Blaming others | Refocusing on planning |
| Refocusing on planning | Catastrophizing |
| Bridge Centrality | Bridge Centrality |
| Externally-Oriented Thoughts | Externally-Oriented Thoughts |
| Blaming others | Catastrophizing |

According to the results in Figures 1-6, the key nodes of the two groups are collated in Table 1. The results reflect that the key nodes of the two groups are quite different, with the internet addiction group including catastrophizing, rumination, refocusing on planning, and Externally-Oriented Thoughts (EOTS), and the non-internet addiction group including positive refocusing, refocusing on planning, blaming others, and EOTS.

Both groups have EOTS as a key node; that is, they both perceive more negative emotions (Moriguchi et al., 2007). More importantly, the cognitive emotion regulation strategies chosen by the two groups are completely different when faced with negative emotions. The addiction group used more negative strategies, whereas the non-addiction group used more positive strategies.

For the addiction group, the interaction of EOTS, catastrophizing, rumination and other strategies causes individuals to produce and exaggerate negative emotions. Some of these individuals use the internet problematically to bring temporary and superficial pleasure to relieve negative emotions, and after repeated reinforcement and conditioning, they gradually form addictive behavior.

For the non-addiction group, the adaptive strategies such as positive refocusing, refocusing on planning and positive reappraisal can effectively relieve negative emotions (Garnefski et al., 2001). Although blaming others is a negative strategy, it is an important node for the non-addiction group, which may be explained by attribution theory (Weiner, 1993). Following negative events, attribution to internal rather than external causes had a reliable and significant association with increased mental problems (Joiner & Wagner, 1995); that is, attribution to external factors (e.g., blaming others) may be related to less distress when individuals confront negative events because it may function to diffuse an individual’s sense of responsibility for that event, which may relieve stress (O’Connor et al., 2011).

To sum up, all the key nodes in the non-addiction group could effectively relieve the negative emotions. But none of the important nodes found in the addiction group could effectively relieve the negative emotions, thus increasing the possibility of internet access as a means of self-regulation.

Reference

Cao Jianqin, Yang Jinwei, Yang Jun, Yao Dazhi, and Zhou Yuqiu (2010). Reliability and Validity of Internet Addiction Impairment Indexes. *Chinese General Practice* 13, 3903–3905. doi: 10.3969/j.issn.1007-9572.2010.34.026.

Garnefski, N., Kraaij, V., and Spinhoven, P. (2001). Negative life events, cognitive emotion regulation and emotional problems. *Personality and Individual Differences* 30, 1311–1327. doi: 10.1016/S0191-8869(00)00113-6.

Joiner, Jr., T. E., & Wagner, K. D. (1995). Attributional style and depression in children and adolescents: A meta-analytical review. *Clinical Psychology Review,* 15(8), 777–798. <https://doi.org/10.1016/0272-7358(95)00046-1>

Moriguchi, Y., Maeda, M., Igarashi, T., Ishikawa, T., Shoji, M., Kubo, C., et al. (2007). Age and gender effect on alexithymia in large, Japanese community and clinical samples: a cross-validation study of the Toronto Alexithymia Scale (TAS-20). *BioPsychoSocial Medicine* 1, 7. doi: 10.1186/1751-0759-1-7.

O'Connor, N., Kotze, B., & Wright, M. (2011). Blame and accountability 1: understanding blame and blame pathologies. *Australasian psychiatry : bulletin of Royal Australian and New Zealand College of Psychiatrists*, *19*(2), 113–118. <https://doi.org/10.3109/10398562.2011.562296>

Weiner, B. (1993). On sin versus sickness: A theory of perceived responsibility and social motivation. *American Psychologist*, 48(9), 957–965. https://doi. org/10.1037/0003-066x.48.9.957
